# Editorial: Cross-Disciplinary Approaches to Characterize Gait and Posture Disturbances in Aging and Related Diseases

**DOI:** 10.3389/fbioe.2022.888910

**Published:** 2022-04-12

**Authors:** Simone Tassani, Martina Mancini, Egon Perilli, Juan Ramírez

**Affiliations:** ^1^ BCN MedTech, DTIC, Universitat Pompeu Fabra, Barcelona, Spain; ^2^ Department of Neurology, Oregon Health and Science University, Portland, OR, United States; ^3^ Medical Device Research Institute, College of Science and Engineering, Flinders University, Adelaide, SA, Australia; ^4^ Mechanical Engineering Department, Universidad Nacional de Colombia, Medellin, Colombia

**Keywords:** gait, multi-factorial analysis, repeatability, aging, imaging, musculo skeletal model, cognitive disorders, Parkinson

Gait abnormalities can be caused by natural signs of aging and/or by specific diseases. Therefore, any study that aims to analyze gait in the elderly faces the problem of the interaction between, at least, these two factors. Even if gait analysis nowadays is a solid and well-known tool for research, the interpretation of the results is strongly linked to the interdependence among the different aspects that can affect the subjects in analysis. The most obvious solution to this problem is to focus on narrow studies centered on specific techniques or patients’ conditions. Such studies are important, and in the past have led to several methodological advances. However, not considering age-related factors that are relevant for gait and posture analysis can generate misleading and contradictory results. Contemporary research often suffers from a lack of repeatability, inconsistency of results, and confounding of parameters. These problems can be related to the limited cross-disciplinary approaches applied. Specific studies can appear significant when performed individually, but they can lose significance when included in the wider research context in which they belong.

The current research topic presents a Research Topic of studies that tried to cross the borders of current musculoskeletal (MSK) science, setting interdisciplinary goals, merging multiple aspects, and therefore considering the effect that external factors can have over gait and its interpretation.

For instance, sight is known to affect posture, stability, and in general, movement in space, and for this reason, studying a combination of factors covering gait and the visual system can allow early detection of a range of various conditions.

In the work by Lirani-silva et al., as described in this very topic, eye-tracking systems were used during the gait of subjects with mild traumatic brain injury (mTBI). mTBI can result from several mechanisms and at any age; however, falling, which is a major problem in the elderly, clearly belongs to the possible causes leading to mTBI. The authors find a positive interaction between the condition of the patients and the saccade duration, showing how gait speed can be related to saccade time for people with mTBI. The interaction between the visual system and posture is also studied by Kahya et al. The authors present a relation between postural stability and pupillary response, as an index of cognitive workload during postural control in patients with Parkinson’s disease (PD). PD patients showed a higher level of pupillary response, and therefore higher cognitive workload, together with a higher displacement of the center of pressure, related to reduced stability. The work presents a potential tool to understand the neurophysiology underpinning falls and the risk of falls. Finally, the study by Ma et al. showed that patients with idiopathic rapid eye movement sleep behavior disorder, which are at high risk for conversion to PD, can be effectively monitored by measuring gait characteristics, such as range of motion and peak angular velocity of the trunk.

Taken together, the findings presented in this research topic suggest that different patterns of gait and posture can therefore be related to the development of different cognitive limitations and potentially be used for their early detection. In particular, the relation between different stages of Huntington’s disease and gait presented here (Talman and Hiller) describing how gait abnormalities are multifactorial, relating to both motor manifestation and cognitive limitations.

Although the relationship between posture and cognitive condition seems strong, we must not fall into the temptation to believe that a single analysis looking at one single aspect of balance, can discriminate among all kinds of dementia. The study by Mc Ardle et al. showed how inertial sensors can be used to discriminate between Alzheimer’s disease and Parkinson’s disease dementia, but not between the mentioned diseases and dementia with Lewy bodies. Discrimination against control subjects was also possible only for patients with Parkinson’s disease dementia, underlining the potential and limitations of inertial sensor-based posture analysis.

The relation between cognition and fall risk is a broad topic and can be approached from different sides (Zhang et al., 2019). The integrated use of different analysis techniques is interesting and finds an appropriate space in this research topic; the integration of diverse techniques is demonstrated to lead to more insightful results than those obtained by applying the same techniques individually. However, the need for integration also presents difficulties that are specific to the combination of different topics and must be properly studied as a research subject itself.

Imaging is one of the analysis techniques most often associated with movement analysis (Bürki et al., 2017; Maillet et al., 2012). Le Floch et al. present a correlation among cognitive impairments, history of falls, and brain areas identified using voxel-based morphometric analysis of magnetic resonance images (MRI). The authors find that older fallers have larger subvolumes in the bilateral striatum, suggesting an adaptive mechanism to falls in people with neurocognitive decline.

Given the great importance of imaging in the study of age-related diseases and its wide use in biomechanical modeling, also providing some space within this research topic for the study of the possible risks to which MRI operators are exposed to appeared to be important (Gurrera et al.). The study presents the integrated use of modeling of the magnetic field associated with the movement analysis of operators in the MRI environment. As a result the authors identify specific tasks that can be considered safe in such an MRI-related work environment.

MRI and other biomedical imaging techniques are often used for the development of biomechanical patient-specific models (Andreaus and Iacoviello, 2012). By combining imaging and modeling with gait analysis it is possible to further explore variables that can influence the analysis of human movement in the elderly. Here, De Pieri et al. studied hip contact forces (HCFs) in asymptomatic subjects to identify possible abnormalities that might lead to the development of osteoarthrosis (OA). The authors found that higher femoral antetorsion led to significantly higher anteromedial HCFs identifying a possible cause for future degeneration of the cartilage. The study is of particular interest because it stresses how the kinematic characteristics of gait might generate abnormal situations and possibly lead to the development of hip OA. Although morphometrical factors are often mentioned as a possible cause of cartilage degeneration, a study using MRI-based bone geometry coupled with subject-specific MSK modeling identified how the contribution of bone shape to model-derived joint kinematics lacked clinical relevance (De Roeck et al.), while underlining the dominant role of movement over the one of morphology variability. The loads produced by different gait patterns propagate to the tissue and cell level and can initiate, for instance, the degeneration of cartilage in OA subjects. A variety of analysis techniques can be organized in multiple subsequent levels using the output of one model as a boundary condition of the next one. This approach allows researchers to create a top-down multilevel model that can be used to explore the effect of macro-factors over the micro ones. Caravaggi et al. present an original methodology to study the effect of cyclic joint loading on cartilage metabolism, combining biomechanical data and medical imaging with molecular information. The protocol has the potential to be applied to explore molecular pathways in the development of OA. This information on pathways can also be crucial for the selection of OA therapies. Nowadays, the decision to select conservative treatments or surgery is mainly related to the beliefs of the medical doctor treating the patient. The result is that patients with a similar degree of OA can follow very different clinical treatment regimes. There is a need to develop new approaches and metrics that can help clinicians in the selection of the best therapy for a specific patient. As a step in this direction, Tassani et al. have presented a multifactorial approach that allowed the stratification of patients in several groups with comparable knee OA but different clinical characteristics. The work allowed the study of nonlinear interaction among multiple factors and showed how the functionality, in terms of step time, speed, and double stance, might be a better indicator than contact forces and moments, for the identification of the appropriate clinical treatment.

In the context of the integration of multiple models and factors, the study of uncertainty related to MSK models driven by gait analysis is one of the topics presented in this Research Topic (Curreli et al.). The authors present a crucial problem in the typical approach to couple MSK and finite element models (FEMs): The identification and quantification of the uncertainties related to the boundary conditions. Different complex and non-linear models coupled together can interact with each other to create a dynamic and non-linear system. Such models will present high variability of the output despite small variability of the input, thus limiting the repeatability of the results when interactions among factors are not considered. Curreli et al. apply variable inputs to MSK models to study the impact of total knee replacement showing how the different kinematic definitions implemented in the models influence the motion and the load history of the artificial joint.

This Research Topic thus brings together 12 peer-reviewed papers addressing the two main factors that influence the analysis of human motion: the interaction of gait with cognitive conditions and the integration of multiple techniques for gait analysis. As illustrated convincingly in the research collected here, an understanding of the role that factors not directly related to the motor system can have on the final analysis of human motion is highly relevant for the interpretation of our results.

We hope that the reader will find in this Research Topic a useful reference for the state of the art in gait and posture disturbances in aging and age-related diseases.
